# Compartmentalization engineering of yeasts to overcome precursor limitations and cytotoxicity in terpenoid production

**DOI:** 10.3389/fbioe.2023.1132244

**Published:** 2023-02-23

**Authors:** Lifei Chen, Wenhai Xiao, Mingdong Yao, Ying Wang, Yingjin Yuan

**Affiliations:** ^1^ Frontier Science Center for Synthetic Biology and Key Laboratory of Systems Bioengineering (Ministry of Education), School of Chemical Engineering and Technology, Tianjin University, Tianjin, China; ^2^ Georgia Tech Shenzhen Institute, Tianjin University, Shenzhen, China

**Keywords:** compartmentalization, terpenoids, precursor limitation, cell cytotoxicity, metabolic engineering

## Abstract

Metabolic engineering strategies for terpenoid production have mainly focused on bottlenecks in the supply of precursor molecules and cytotoxicity to terpenoids. In recent years, the strategies involving compartmentalization in eukaryotic cells has rapidly developed and have provided several advantages in the supply of precursors, cofactors and a suitable physiochemical environment for product storage. In this review, we provide a comprehensive analysis of organelle compartmentalization for terpenoid production, which can guide the rewiring of subcellular metabolism to make full use of precursors, reduce metabolite toxicity, as well as provide suitable storage capacity and environment. Additionally, the strategies that can enhance the efficiency of a relocated pathway by increasing the number and size of organelles, expanding the cell membrane and targeting metabolic pathways in several organelles are also discussed. Finally, the challenges and future perspectives of this approach for the terpenoid biosynthesis are also discussed.

## Introduction

Terpenoids are a large family of more than 8,000 natural compounds with a wide range of applications, such as pharmaceuticals, agrochemicals, food additives and biofuels. All terpenoids are derived from isopentenyl diphosphate (IPP) and dimethylallyl diphosphate (DMAPP), *via* the larger prenyl diphosphate compounds farnesyl diphosphate (FPP), geranyl diphosphate (GPP), or geranylgeranyl diphosphate (GGPP), which represent the universal pool of precursors for terpenoid biosynthesis. According to the number of isoprene units (Zhang and Hong, 2020), terpenoids are classified as monoterpenoids (C10), sesquiterpenoids (C15), diterpenoids (C20), triterpenoids (C30), and tetraterpenoids (carotenoids, C40). In recent years, the rapid development of metabolic engineering and synthetic biology has led to the development of alternative approaches meet the increasing demand for terpenoids (Chen et al., 2018). In eukaryotic cells, common strategies include increasing the supply of acetyl-CoA (Chen et al., 2013), upregulation of the mevalonate (MVA) pathway (Ro et al., 2006), and engineering that improves the supply of cofactors (Chen R. et al., 2022), some of which are common to all terpenoids (Zhang et al., 2022).

**FIGURE 1:**
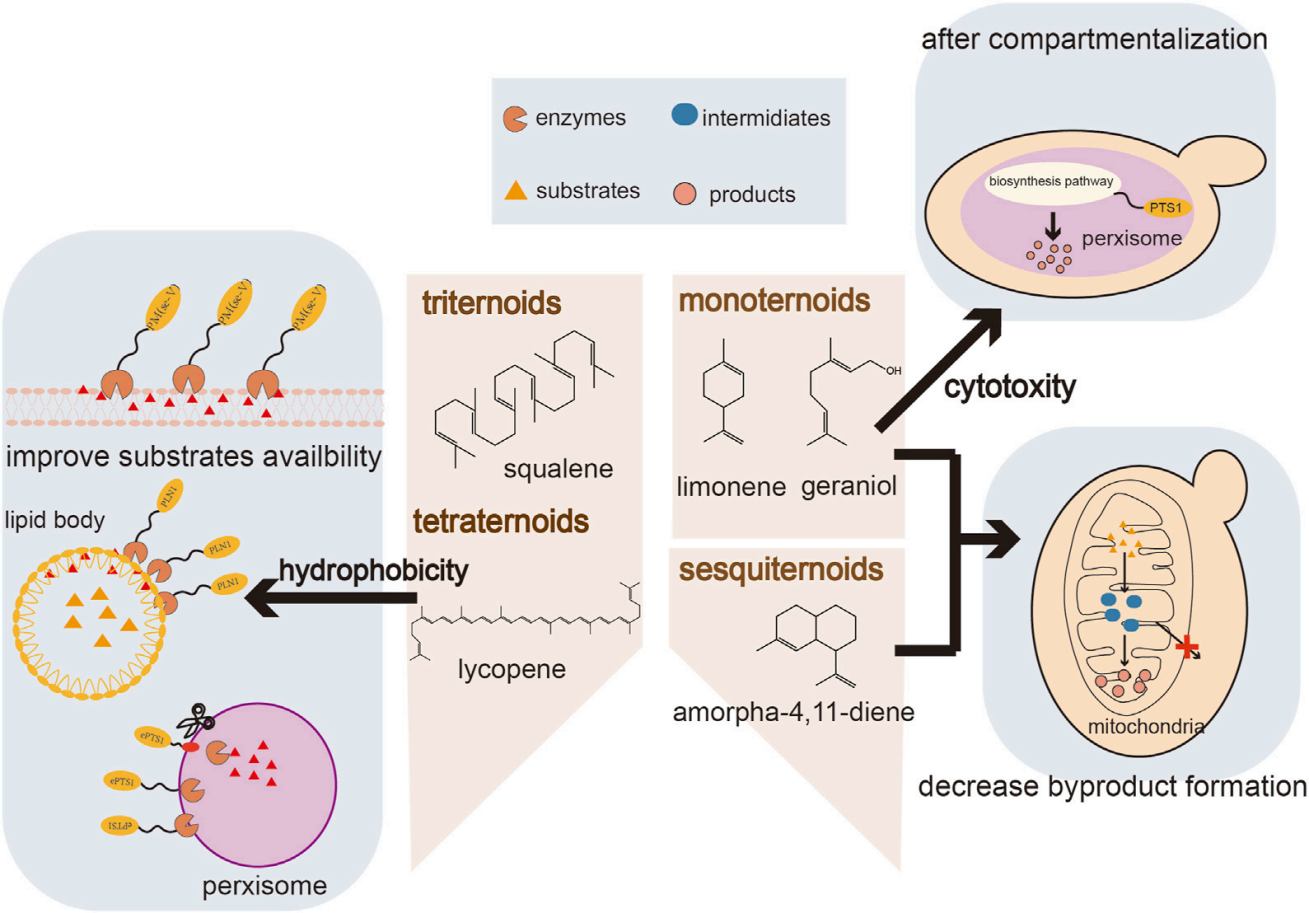
An overview of compartmentalization strategies for terpenoid production.

In spite of the common upstream pathway, the enormous structural diversity of terpenoids presents many unique challenges for their biosynthesis, necessitating specific optimization strategies for different types of terpenoids (Figure 1). The insufficient precursor supply is an important factor restricting the production of monoterpenes and sesquiterpenes (Moser and Pichler, 2019). GPP, the precursor of monoterpenes, is also an intermediate of the pathway leading to FPP. Once synthesized, GPP is rapidly converted by FPP synthase (ERG20) which limits the GPP pools and blocks the production of monoterpenoids (Ignea et al., 2014). Likewise, FPP is channeled toward sterol biosynthesis by ERG9, which is essential for cell survival and cannot be completely knocked out. In addition, the cytotoxicity of monoterpenes can have a negative effect on the producing hosts and thereby limits further improvement of monoterpenoid production (Zhu K. et al., 2021). Triterpenes and tetraterpenes are large molecules that accumulate intracellularly in specific subcellular compartments, while some terpenoids are not suitable for intracellular accumulation due to their cytotoxicity. The storge capacity of organelles with hydrophobic environments are essential for the terpenoid production. To overcome these challenges caused by terpenoids, strategies involving the optimization of the expression of key enzymes, tolerance engineering and transporter engineering have been proposed to reduce precursor limitations and cytotoxicity, have resulted in significant improvements in terpenoid production (Peng et al., 2017; Zhang et al., 2017).

Strategies to control the subcellular arrangement of metabolic enzymes are a promising approach for resolving the challenges of terpenoid production mentioned above. Eukaryotic cells contain several specific organelles, including mitochondria, peroxisomes, endoplasmic reticulum (ER), lipid bodies (LDs) and the cell membrane (Dyall et al., 2004). All these organelles possess a complex structure, whereby the specific cofactors, metabolites, and unique physicochemical environments of these organelles offers different conditions for different metabolic pathways (Cao et al., 2020). Some organelles, such as mitochondria, peroxisomes and LDs, have phospholipid membranes, which can increase the local concentration of substrates and enzymes inside the smaller organelle compartments. Compartmentalization also blocks competing reactions and reduces the toxicity of intermediates or substrates by organellar insulation (Gao and Zhou, 2019). The production of terpenoids involves complex metabolic compartmentalization, which partly explains the low yield in terpenoid production achieved to date (Basiony et al., 2022), especially using heterologous yeasts. In heterologous biosynthesis in yeasts, enzymes re-locate to specific organelles and some terpenoids accumulate in membrane-like structures such as LDs. The enzymes and its substates are distributed in different organelles in yeast, which result in the separation of enzyme and substate. (Zhu Z-T. et al., 2021). Targeting the biosynthetic pathway to the same organelle where the desired products are stored enhances the biosynthesis of terpenoids. Subcellular compartmentalization for rewiring of metabolic flux can overcome serveral tackles and enhances the productivity of terpenoids pathways.

Many studies have harnessed subcellular compartments to improve terpenoid biosynthesis in yeasts (Hammer and Avalos, 2017; Yocum et al., 2021; Jin et al., 2022), as shown in Table 1. The physiological properties of various organelles as well as their benefits and drawbacks for organelle compartmentalization in terpenoid production (Jin et al., 2022), which will not be discussed below. In this review, we summarize the characteristics of monoterpenoids, sesquiterpenoids and tetraterpenoids and focuse on their unique requirements for improving terpenoid production through organelle compartmentalization. In addition, we provide an overview of recent progress in the modification of organellar morphology for subcellular compartmentalization in terpenoid production.

**TABLE 1:** Organelle compartmentalization and optimization of terpenoids production.

	Products	Strategy	Organism	Compartment	Title	Modification of organelle	Reference
monoterpene	Geraniol, R-(+)-limonene, CBGA	• Construct a monoterpenoids producing platform in peroxisome	*Saccharomyces cerevisiae*	Peroxisome cytoplasm	5.5 g/L (geraniol) 2.6 g/L	—	Dusséaux et al. (2020)
		• Insulate the GPP from competing pathway					
		• Used peroxisome as detoxifying organelle					
	Nepetalactol geraniol	• Target geraniol biosynthetic pathway to the mitochondria	*Saccharomyces cerevisiae*	mitochondria	227 mg/L^a^	—	Yee et al. (2019)
		• Protect the GPP pools from consumption by the cytosolic ergosterol pathway					
	Linalool	• Dual mitochondria and cytoplasm engineering in linalool production	*Saccharomyces cerevisiae*	mitochondria	23.45 mg/L^a^	—	Zhang et al. (2020)
		• Enhance the GPP pools for linalool production					
	Isoprene	• Make full use of acetyl-CoA both in cytoplasm and mitochondrial	*Saccharomyces cerevisiae*	Mitochondria cytoplasm	2,527 mg/L	—	Lvet al. (2016)
	Sabinene	• Utilize the mitochondria and cytoplasm GPP pools	*Saccharomyces cerevisiae*	Mitochondria cytoplasm	154.9 mg/L^a^	AIM25, FIS1, LSB3, MBA1	Jia et al. (2020)
		• Overexpression of mitochondria-related genes to improved sabinene production					
	Geraniol	• Target geraniol biosynthetic pathway to peroxisome	*Saccharomyces cerevisiae*	peroxisome	2.75 mg/L^a^	pex30, pex31, atg36	Gerke et al. (2020)
		• Reduce the cell toxicity					
sesquiterpene	Valencene amorphadiene	• Co-locate FDPS and sesquiterpenes synthases to the mitochondria	*Saccharomyces cerevisiae*	mitochondria	1.5 mg/L^a^ (Valencene) 20 mg/L^a^	—	Farhi et al. (2011)
	α-santalene	• Reconstruct the whole MVA pathway in mitochondria to harness the precursor pools	*Saccharomyces cerevisiae*	mitochondria	41 mg/L^a^	—	Dong et al. (2021)
	amorpha-4,11-diene	• Harness the mitochondria acetyl-CoA for amorpha-4,11-diene production	*Saccharomyces cerevisiae*	mitochondria	427 mg/L^a^	—	Yuan and Ching (2016)
		• reduce loss of FPP to cytosolic competing pathways					
	α-Humulene	• Utilize the native acetyl-CoA pools in peroxisome	*Yarrowia lipolytica*	peroxisome	3.2 g/L	—	Guo Q et al. (2021)
Triterpene	Squalene	• Dual cytoplasmic-peroxisomal engineering for squalene production	*Saccharomyces cerevisiae*	Peroxisome cytoplasm	11 g/L	—	Liu et al. (2020)
		• Dual MVA pathway in mitochondria and cytoplasm to enhance squalene production	*Saccharomyces cerevisiae*	Mitochondria cytoplasm	21.1 g/L	—	Zhu Z-T et al. (2021)
	ginsenoside	• Target protopanaxadiol synthase (PPDS) to LDs	*Saccharomyces cerevisiae*	lipid droplets	5 g/L	GPD1, PAH1, DGAT1, SEI1	Shi et al. (2021)
		• Increase the volumes of lipid droplets					
	protopanaxadiol	• Construction of the protopanaxadiol pathway in peroxisome	*Saccharomyces cerevisiae*	peroxisome	4.1 ± 0.2 mg/L^a^	pex11, pex34, atg36	Choi et al. (2022)
		• optimization of peroxisome proliferation					
	Squalene protopanaxadiol	• Overexpress ER size regulatory factor to increase the production of squalene and protopanaxadiol	*Saccharomyces cerevisiae*	endoplasmic reticulum	634 ± 11 mg/L^a^(squalene)	INO2	Kim et al. (2019)
tetraterpene	Lycopene	• Overexpressed key genes associated with fatty acid synthesis and TAG production and regulate lipid-droplet size to increase lycopene accumulation	*Saccharomyces cerevisiae*	lipid droplets	2.37 g/L	PAH1, DGA1, ACC1, OLE1, FLD1	Ma et al. (2019)
		• Target lycopene pathway to peroxisome	*Pichia pastoris*	peroxisome	73.9 mg/L^a^	—	Bhataya et al. (2009)
	canthaxanthin	• Introduce the β-carotene ketolase variant OBKTM29 to the plasma membrane	*Saccharomyces cerevisiae*	plasma membrane	1.44 g/L	—	Chen M et al. (2022)
	Lutein	• For enforcing metabolic flux towards α-carotene, re-locatingε-cyclase to the plasma membrane	*Saccharomyces cerevisiae*	plasma membrane	110.4 μg/L	—	Bian et al. (2021)
	astaxanthin	• Target the astaxanthin pathway simultaneously to lipid body, endoplasmic reticulum and peroxisome	*Yarrowia lipolytica*	lipid body, endoplasmic reticulum and peroxisome	858 mg/L	—	Ma et al. (2021)

^a^ Fermentation at the shake flask level.

## Monoterpenoids and sesquiterpenoids

### Enhancing the supply and utilization of GPP and FPP

**FIGURE 2:**
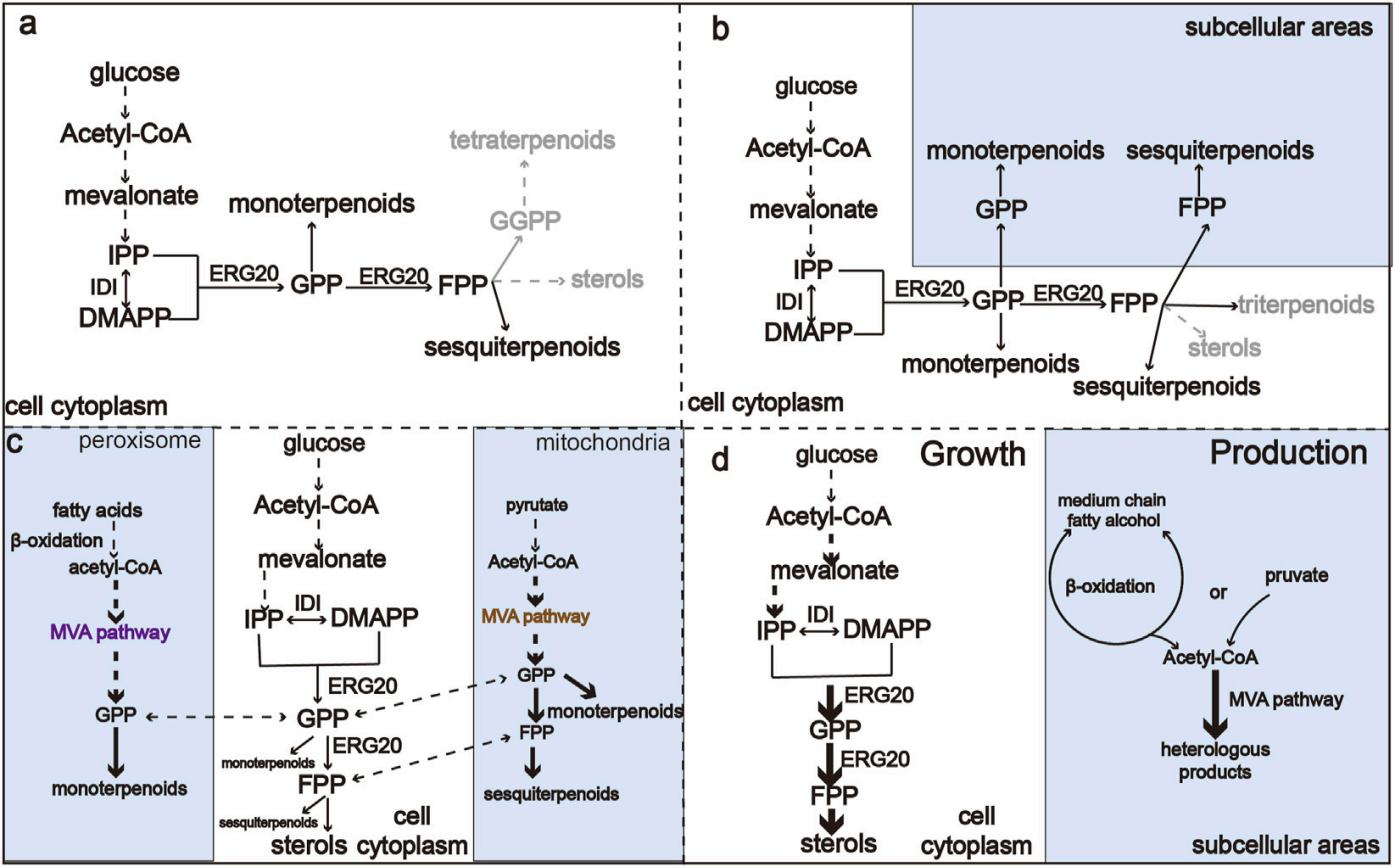
An overview of metabolic regulation of monoterpenoid and sesquiterpenoid biosynthesis pathways. **(A)** Monoterpenoid and sesquiterpenoid biosynthesis pathway in the cytoplasm. **(B)** Harnessing GPP and FPP in both cytoplasm and organelles to improve the production of monoterpenoids and sesquiterpenoids. **(C)** Harnessing acetyl-CoA in organelles to improve the production of monoterpenoids and sesquiterpenoids. **(D)** Engineering compartmentalization for orthogonal biosynthesis of monoterpenoids and sesquiterpenoids to reduce competing reactions.

The difficulty in creating a sufficient precursor pool of GPP and FPP limits the biosynthesis of all monoterpenoids and sesquiterpenoids (Lei et al., 2021). The traditional monoterpenoid and sesquiterpenoid production processes utilize cytoplasmic GPP and FPP (Figure 2A), which is generally limited. However, additional pools of GPP and FPP are distributed in different organelles. Inspired by the concept that organelles of eukaryotic cells can be harnessed to reconstruct metabolic pathways and thereby increase the availability of precursors (Hammer and Avalos, 2017). Targeting the enzymes or the whole biosynthesis pathway to the organelles can effectively optimize monoterpene and sesquiterpene production (Figure 2B). Optimal utilization of GPP and FPP pools distributed in yeast organelles provides a method for microbial overproduction of monoterpenes and sesquiterpenes (Jia et al., 2020). To harness the pool of FPP in mitochondria, Farhi et al. (2011) targeted FPP synthase and sesquiterpene synthase in mitochondria, achieving an eight- and 20-fold improvements in the production of valencene and amorphadiene, respectively. Upstream of FPP and GPP, acetyl-CoA is the central precursor of the MVA pathway for the biosynthesis of terpenoids, and an insufficient supply of acetyl-CoA limits the metabolic flux toward desired compounds (Krivoruchko et al., 2015). As sites of the β-oxidation of fatty acids, peroxisomes can supply ample acetyl-CoA (van der Klei and Veenhuis, 1997). Similarly, mitochondria have nearly 20–30 times higher acetyl-CoA content than the cytosol (Duran et al., 2020). As shown in Figure 2C, rational organelle compartmentalization can greatly improve the utilization efficiency of the acetyl-CoA pools in different organelles (Son et al., 2022a; Lu et al., 2022). Dong et al. (2021) compartmentalized the whole MVA pathway into mitochondria, leading to a 3.7-fold improvement in the production of α-santalene.

In addition to increasing the supply of precursors to enhance the production of mono- and sesquiterpenes, inhibiting competing pathways is also a major strategy to increase the production of desired compounds (Mai et al., 2021). The FPP-derived squalene and native sterol biosynthesis pathways are the main competing pathways of monoterpenoid and sesquiterpene synthesis. However, these pathways are essential for the fluidity of the yeast cell membrane and host growth (Fischer et al., 2011), which limits heterologous monoterpene and sesquiterpene production (Peng et al., 2017). A commonly used method to reduce the competing reactions is to construct and screen mutants of ERG20 (Zhang et al., 2020), degrade the rate-limiting enzymes ERG20 and ERG9 (Peng et al., 2018), or establish an orthogonal pathway that uses neryl diphosphate (NPP) as an alternative substrate (Ignea et al., 2019). Due to the necessity of ergosterol for cells, the heterologous pathway affects cellular function due to ubiquitous metabolic interactions. The regulation of metabolic fluxes is crucial to balance growth and production of the desired molecule. However, metabolic rewiring faces many biological challenges that affect the growth and fitness of the hosts (Zhu Z-T. et al., 2021). A method to overcome this obstacle would be to minimize the interactions between heterologous pathway and native metabolism (Pandit et al., 2017; Ignea et al., 2019). This can be achieved by constructing orthogonal pathways through subcellular compartmentalization (Figure 2D). For example, targeting the MVA biosynthesis pathway and the product biosynthesis pathway toorganelles can separate chassis growth from metabolite production. In addition, organelles such as peroxisome and mitochondria can isolate the intermediates from competing reactions, thus increasing the GPP or FPP pool by avoiding consumption by cytoplasmic enzymes (Yuan and Ching, 2016; Yee et al., 2019; Yee et al., 2019; Zhang et al., 2020). By introducing the complete MVA pathway into the peroxisome, Dusséaux et al. (2020) achieve up to a 125-fold increases in the production of geranyl diphosphate-derived compounds compared to the cytosolic pathway. This strategy can be used to produce GPP- or FPP-derived compounds in eukaryotic cells without noticeable effects on strain fitness or viability. Metabolic rewiring has become a promising strategy for enhancing terpenoid production in yeasts.

### Reducing the cytotoxicity of monoterpenoids and sesquiterpenoids

The high toxicity of monoterpenoids remains a challenging issue (Erdogan and Ozkan, 2017). Notably, monoterpenoids not only interfere with cell walls and organellar membranes by altering membrane fluidity, structural membrane integrity, and membrane composition, but also induce oxidative stress, and bring more toxic monoterpene hydroperoxides (Brennan et al., 2013; Moon et al., 2020). As shown in Table 2, traditional strategies such as transporter engineering (Hu et al., 2020), *in situ* product extraction (Rolf et al., 2020), and tolerance engineering can improve the tolerance of strains to monoterpenes and reducetoxicity (Brennan et al., 2015; Jezierska and Van Bogaert, 2017; Zhu K. et al., 2021). To address the metabolic toxicity of monoterpenoids, intracellular compartmentalization of their biosynthetic pathways is commonly used to insolate the monoterpenoids into specific organelles (Kulagina et al., 2021). The monoterpene indole alkaloids produced in *Catharanthus roseus* were distributed into several organelles to relieve cell toxicity, which provides new insight into how subcellular compartmentalization can enhance the metabolic flux towards the biosynthesis of target compounds (Courdavault et al., 2014; Guirimand et al., 2020). Peroxisomes are not essential for cell growth (Sibirny, 2016), and are also detoxifying organelles (Breitling et al., 2002). Accordingly, the localization of biosynthetic enzymes or whole biosynthetic pathways to peroxisomes may insulate the toxic components away from the host cell cytosol. The peroxisome can be used as a detoxifying organelle for the synthesis of monoterpenes, including limonene, geraniol, α-pinene, sabinene and camphene (Dusséaux et al., 2020). Dusséaux et al. (2020) constructed peroxisome microfactorties able to accelerate recovery growth by the reducing cytotoxicity of monoterpene products. Gerke et al. (2020) targeted the geraniol biosynthetic enzymes to peroxisomes and improved the geraniol tolerance of the yeast cells to reduce product toxicity, resulting in an 80% increase in the geraniol titer. A combination of traditional metabolic strategies and organelle compartmentalization to alleviate product cytotoxicity may provide new insights.

**TABLE 2:** Comparison of reducing cytotoxicity strategies of monoterpenoids production.

	Strategies	Advantage	Shortage	References
Traditional strategies	Transporter engineering (Isolation of target chemicals from cells through membrane protein)	High transport efficiency; High substrate specificity	Challenges to obtain non-natural transporter by protein engineering	Langevin et al. (2020)
	Extraction of *in situ* products (Transport of inhibitory products into the organic phase)	Simple and effective; Not reliance genetic engineering	The organic phase is not suitable for some water-soluble products	Brennan et al. (2012)
	Tolerance engineering (Modulation of the cellular physiology to tolerate toxic products)	Not rely on genetic engineering; Easy operation	Time consuming and labour-intensive	Li et al. (2021)
Organelle compartmentalization	(Targeting of biosynthesis pathway to organelles)	Increases the concentration of enzymes and substrates; Reduces toxicity effectively and relieves bypass competing pathway monoterpene hydroperoxides	somtimes poor targeting of desired protein into organelle	Grewal et al. (2021)

## Triterpenoids and tetraterpenoids

### Reducing cytotoxicity of triterpenoids and tetraterpenoids

**FIGURE 3:**
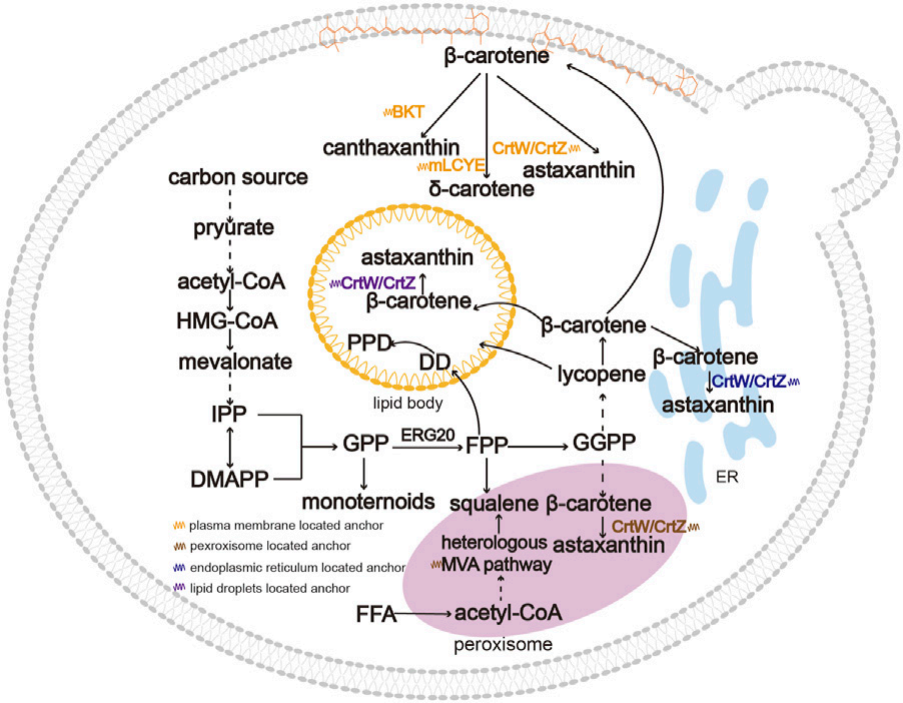
An overview of subcellular compartmentalization for the biosynthesis of triterpenoids and tetraterpenoids. Due to their hydrophobicity, triterpenoids and tetraterpenoids are generally sequestered in specific organelles such as LDs, peroxisomes, ER and plasma membrane. Product distribution in strains reduces cytotoxicity and causes spatial separation between substates and products. Moreover, targeting the pathway to more than one compartment can increase the product titer compared to single subcellular compartmentalization. Abbreviations: BKT, β-carotene ketolase; mLCYE, lycopene ε-cyclase.

The long-chain triterpenoids and tetraterpenoids are highly polyunsaturated lipophilic hydrocarbons. Similar to triacylglycerols (TAGs) and sterol esters (SEs), triterpenoids and tetraterpenoids are generally sequestered in specific subcellular areas because of their hydrophobicity (Spanova et al., 2010; Wei et al., 2018). As shown in Figure 3, different organelles such as LDs, peroxisomes, ER and plasma membrane can be used as terpenoid reservoirs due to their similar hydrophobicity (Cunningham and Gantt, 1998; Lee et al., 2009). Cytotoxicity is caused by the excessive accumulation of triterpenoids and tetraterpenoids in the host. The plasma membrane provides storage space for the accumulation of heterologous hydrophobic target compounds (Wu et al., 2017), such as carotenoids (Liu et al., 2016). Targeting the key enzymes to the plasma membrane can effectively alleviate the toxicity of hydrophobic compounds and increase production (Bian et al., 2021; Chen M. et al., 2022). Ye et al. (2018) fused a β-carotene ketolase (CrtW) and β-carotene hydroxylase (CrtZ) and targeted the fusion protein to the cell membrane, which resulted in a 215.4% increase in astaxanthin production. By regulating steady-state fluxes and the availability of intermediate metabolites, compartmentalization engineering in yeasts can significantly shorten the distance between the enzyme and the substrate and increase local concentration of the enzyme and the substrate, thereby improve the product yield (Valm et al., 2017).

### Providing a suitable environment for the production of triterpenoids and tetraterpenoids

Organelles such as LDs and peroxisomes can provide a suitable storage environment to relive the cytotoxicity of triterpenoids and tetraterpenoids. LDs and peroxisomes are involved in cellular lipid metabolism, among which peroxisomes play a vital role in α- and β-oxidation of fatty acids. These compartments can provide different intracellular environments for enzyme catalysis, such as adequate substrate supply and cofactor balance (Gao and Zhou, 2019). Peroxisomes are enclosed by a single membrane and are speculated to be an additional cellular storage space for lipophilic products in eukaryotic microbial hosts, acting as an advantageous dynamic depot for storing hydrophobic compounds like squalene (Liu et al., 2020). Liu et al. (2020) compartmentalized the entire squalene synthesis pathway into peroxisomes, followed by dual modulation through cytoplasmic and peroxisomal engineering, which improved the squalene titer to 1.7 g/L. Squalene is an important precursor for triterpenoid biosynthesis, and re-locating triterpene synthases to peroxisomes would be a promising solution for triterpenoid production. Recently, the importance of LDs for the storage of hydrophobic carotenoids was highlighted (Larroude et al., 2018; Bu et al., 2022). LDs serve as specialized platforms for lipid metabolism and storage, with a phospholipid monolayer in which molecules are transported in and out of a LDs (Henne et al., 2020). Ma et al. (2019) observed lycopene accumulated in LDs of *S. cerevisiae*. The hydrophobic compounds are distributed in different parts of LDs due to their different structures (Son et al., 2022b). Adjusting organelle morphology can be a useful strategy for increasing terpenoid production, which will be introduced in detail below. Therefore, the selection of organelles that are naturally lipophilic or hydrophobic may be more conducive to the production of triterpenoids and tetraterpenoids.

### Reducing spatial separation between enzyme and product

The triterpenoids and tetraterpenoids always accumulate intracellularly in specific organelles, while enzymes locate in other organelles such as the cytoplasm or ER; thus, the spatial distance between the enzyme and its product may influence the yield of the resulting products. Breaking the barrier between the enzyme and the substrate can reinforce metabolic flux and improve biosynthesis efficiency. The accumulation of sterol metabolites (e.g., zymosterol) in LDs prevents their conversion by enzymes located in ER. Guo et al. reconstituted the post-squalene pathway in LDs and revealed an effective method of pathway compartmentalization (Guo X-J. et al., 2021). The location of enzymes of biosynthesis pathways in membrane-enclosed LDs or peroxisomes can significantly enhance the metabolic flux and promote product titers. Shi et al. (2021) co-localized the pathway enzymes in LDs to eliminate the separation between the substrate dammarenediol-II (DD) and protopanaxadiol synthase (PPDS) of the PPD biosynthesis pathway, which increased the conversion rate of DD to PPD from 17.4% to 86.0%. Compartmentalized reconstitution is an effective way of providing adequate storage and improving the output of triterpenoids and tetraterpenoids.

## Modification of organellar morphology

### Lipid droplets

**FIGURE 4:**
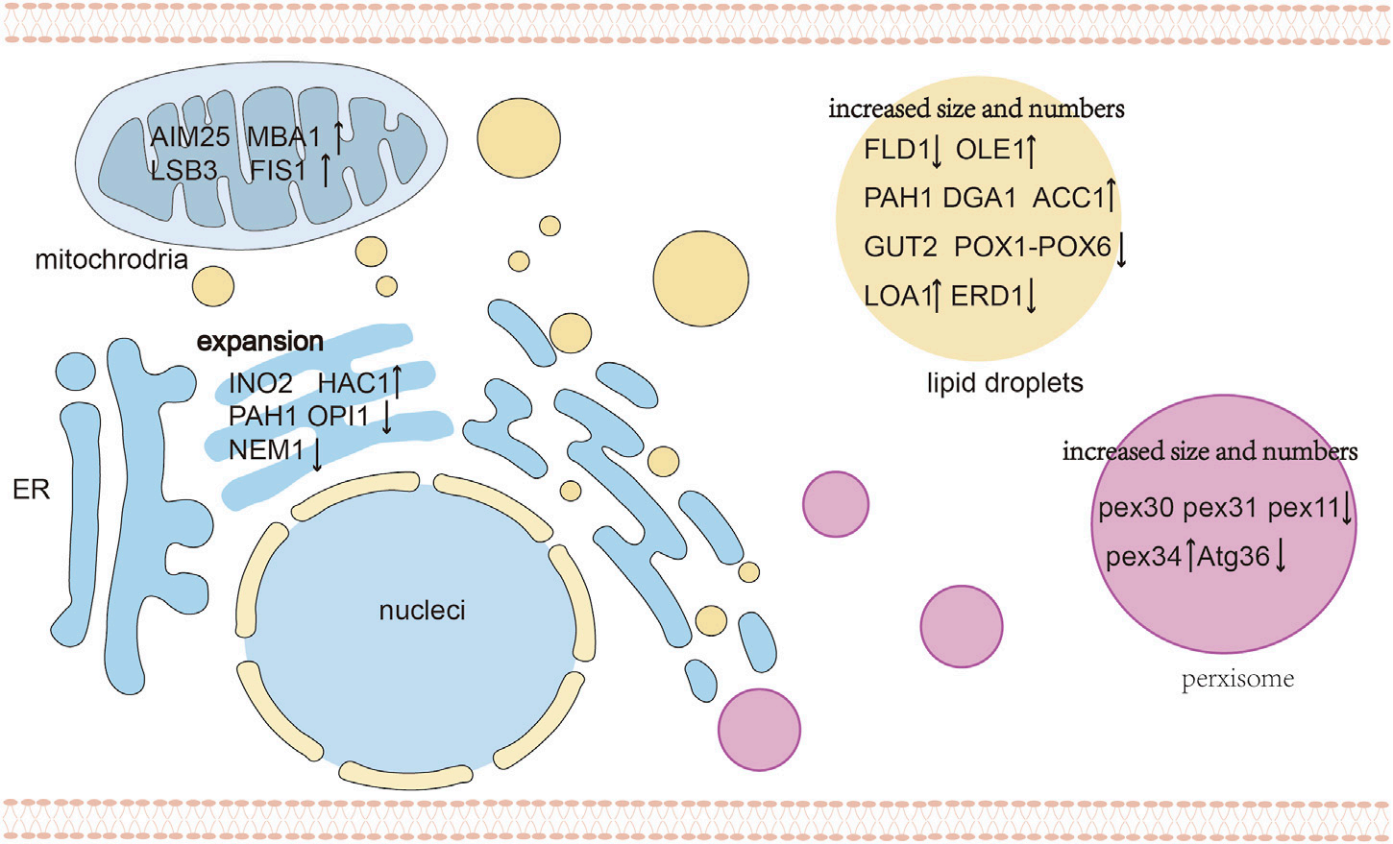
An overview of organelle morphology engineering. The target genes of different organelles are shown to regulate organelle morphology, including increased size and number of LDs and peroxisomes, expansion for ER and engineering mitochondrial physiology. Abbreviations: FLD1, seipin; OLE1, fatty acid desaturase; GUT2, G3P dehydrogenase; POX1-6, acyl-CoA oxidases 1 through 6; PEX11, PEX30, PEX32, PEX34, peroxisome-population-regulated proteins; ATG36, autophagy-related protein; OPI1, negative regulator of phospholipid biosynthesis; NEM1, nuclear envelope morphology protein 1; HAC1, Transcriptional activator; ↑ Gene overexpression; ↓ Gene knockdown.

Subcellular compartmentalization leads to the co-accumulation and co-localization of enzymes and products in organelles. Son et al. reported that flexible lipids such as squalene with conjugated *π* bonds are readily dispersed among TAGs, and squalene production can be enhanced by expanding the volume of the LD. Similarly, rigid lipids such as zeaxanthin and β-carotene are retained between TAGs, and the production of zeaxanthin was enhanced by increasing the LD surface area within the cell (Son et al., 2022b). Thus, LDs are suitable organelles for storing triterpenoids and tetraterpenoids, while strategies that control the size and the number of organelles influence their storage capacity (Figure 4). To achieve a high production of lipophilic terpenoids, enhanced the TAG biosynthesis and reduced TAG degradation have been applied to increase LD volume, thereby promoting intracellular terpenoids accumulation (Wei et al., 2018; Ma et al., 2019). Diacylglycerol acyltransferases (DGATs) and phosphatidate phosphatase (PAP) are crucial enzymes that catalyze the ultimate step of TAG synthesis (Adeyo et al., 2011). Overexpression of DGA1 increase lipid content in yeasts, expanding the intracellular storage pool and significantly promoting the accumulation of lycopene (Ma et al., 2019), squalene (Wei et al., 2021) and other terpenoids (Yu et al., 2020). Shi et al. (2021) targeted the limiting enzymes PPDS to LDs and overexpressed DGA1 to increase LD volume, after which the CK titer of the resulting strain reached 5 g/L in 5 L of fed-batch fermentation. Relevant genes that encoding phosphatidic acid phosphatase (PAH1), acetyl-CoA carboxylase (ACC1), and fatty acid desaturase (OLE1) can also regulate TAG biosynthesis (Karanasios et al., 2013; Shi et al., 2014). In addition to LD volume, regulating the number of LDs to increases the monolayer surface is another important strategy affecting terpenoid production (Shi et al., 2021). Overexpressing the *LOA1* gene and deleting *ERD1* can induce the stress response of the ER and stimulate LD formation, which leads to large numbers of LDs (Ayciriex et al., 2012; Teixeira et al., 2018).

### Peroxisome

Similar to LDs, alternative methods that manipulate the number and volume of peroxisomes can effectively enhance terpenoid production. By regulating the specific surface area, quantity and size of the organelles, it is possible to enhance the content of its host localization proteins and pathway metabolites, which can further increase the flux of the localized pathway. The morphology of peroxisomes is regulated by peroxins (PEX) and dynamin-related proteins (DRPs), as well as the autophagy (ATG) protein family, which are responsible for peroxisome biogenesis, fission and pexophagy, respectively (Islinger et al., 2018). Most PEX genes are involved in the import of matrix proteins (Heiland and Erdmann, 2005), and the remaining PEX genes are involved in regulation of the abundance and size of peroxisomes (Vizeacoumar et al., 2003; Vizeacoumar et al., 2004; Tower et al., 2011; Yofe et al., 2017). To maximize the storage capacity of the peroxisome membrane of *S. cerevisiae*, Choi et al. increased the expression of *PEX34*, deleted the *PEX11* and *ATG36* gene, after which they introduced a heterologous protopanaxadiol pathway (Choi et al., 2022). Deletion of *PEX11* generated enlarged and clustered peroxisomes, while *PEX34*, overexpression of *PEX34* and deletion of *ATG36* significantly increased the number of peroxisomes. The deletion of *PEX30*, *PEX31*, and *ATG36* in a geraniol producing strains with biosynthetic enzymes targeted to the peroxisome resulted in a larger number of peroxisomes and increased the geraniol titer by 80% (Gerke et al., 2020).

### ER and membrane engineering

The expanded membranes of the engineered strains can also be used for additional storage of hydrophobic compounds. Expansion of the ER can increase its capacity to promote the expression of ER-localized proteins and boost terpene accumulation (Kim et al., 2019). Furthermore, suppressing the dephosphorylation and activation of PAH1 can enhance membrane protein productivity and increase the surface area of the ER membrane (Ju et al., 2022). The disruption of PAH1 resulted in a decreased number of LDs, accompanied by the accumulation of neutral lipids in the ER (Adeyo et al., 2011). Similarly to PAH1, overexpression of INO2 in yeast increased its capacity to synthesize endogenous and heterologous ER-associated proteins, leading to a significantly increased squalene titer of 634 mg/L (Kim et al., 2019). Engineering the membrane morphology and improving the membrane synthesis pathway can also increase the stability of membrane-anchored biosynthetic pathways and enhance terpenoid production. A Tsr-augmented recombinant *Escherichia coli* was able to extend the membrane network, enhance the squalene storage and improved the squalene titer to 712 mg/L (Meng et al., 2020). The native *frd* operon and UncF protein also induce membrane invagination and increase the membrane lipid volume (Elmes et al., 1986; Arechaga et al., 2000). These findings suggest that membrane engineering has achieved good progress in terpenoid production in *E. coli*, and suggest a great potential for the utilization of similar mechanisms to modify the membrane area of yeasts. Membrane engineering is a rapidly developing field with great potential for organelle compartmentalization and metabolic engineering.

### Mitochondria

Yeast mitochondria are highly dynamic in size, activity, number, and surface area depending on growth conditions, carbon source and metabolic state (Okamoto and Shaw, 2005). The engineering of mitochondria to increase their activity, morphology, number, and localization can increase the activity of mitochondrial biosynthetic pathways. Jia et al. (2020) overexpressed mitochondria-related proteins including *FIS1* for mitochondrial division, *LSB3* for mitochondrial motility, *MBA1* and *AIM25* encoding other functional proteins to enhance the compartmentalized pathways, and the *AIM25* gene boosted the sabinene titer to 154.9 mg/L. The understanding of mitochondrial physiology and metabolism could be useful to expand the applicability of mitochondrial engineering and realizing its full potential (Ferramosca and Zara, 2021).

### Anchoring enzymes simultaneously to several organelles

Organelles such as mitochondria, ER, LDs, and peroxisomes provide different advantages in regulating metabolic fluxes. To further make use of precursors and promote the dynamic balance between different organelles, dual or triple metabolic regulation of the terpenoid synthesis pathway in different subcellular areas could open new possibilities for the production of terpenoids (Lv et al., 2016; Zhu Z-T. et al., 2021; Guo et al., 2022). Comprehensive utilization of multiple organelles can not only enhance the utilization of the limited hydrophobic storage environment, but can also make full use of hydrophobic substrates such as carotene located in different organelles. Ma et al. (2021) expressed the astaxanthin pathway in LDs, endoplasmic reticulum and peroxisomes, and the best strain ultimately produced 858 mg/L of astaxanthin in fed-batch fermentation. Zhu et al. compartmentalized the MVA pathway to mitochondria to improve cell growth and synthesized squalene in the cytoplasm to improve production (Zhu Z-T. et al., 2021). The simultaneous localization of terpenes in different organelles can serve as a promising strategy for optimizing the biosynthetic pathway module and cell growth in parallel.

## Conclusion and perspectives

Anchoring the key enzymes or the MVA pathway to specific organelles by localization tags can yield substantial improvements in terpenoid production. In this review, we have systematically analyzed successful approaches using organelle compartmentalization applied to overcome problems associated with the production of different terpenoids, including inadequate supply of GPP/FPP pools and other substrates, cytotoxicity of terpenoids, insufficient storge space, and special challenges of tetraterpenoids. However, more work to improve terpenoid production is still needed in the future. Diterpenoids are one of the most important families of bioactive compounds, and compartmentalization is still limited in diterpenoid production in yeasts. Li et al. (2019) compartmentalized taxadiene synthase, taxadiene-5ahydroxylase and cytochrome P450 reductase to the chloroplast of *Nicotiana benthamiana* to improve the supply of precursor for taxadiene synthesis. The accumulation of different diterpenoids has temporal and spatial specificity, and compartmentalization of key enzymes and biosynthetic pathway of diterpenoids is a promising alternative to current methods.

As the most extensively studied organelles in cellular physiology, the engineering of mitochondria, peroxisomes, ER, LDs, and plasma membranes has already led to great progress in terpenoid production. However, future studies should target new organelles like the Golgi apparatus and the vacuole whose potential to improve terpenoid production remains unexplored. For secologanin biosynthesis in *C. roseus*, geraniol from the MEP pathway is directed toward the vacuole following a hydroxylation reaction by *G10H* (Verma et al., 2012). Some terpenoids and relevant enzymes are naturally located in the vacuoles (Guirimand et al., 2011), which may provide an alternative option for terpenoid production as a detoxification compartment and proper site for enzyme catalysis. In eukaryotic cells, cofactors are distributed in different subcellular compartments, which necessitates systematically engineering the supply and recycling of cofactors to couple compartmentalized cellular metabolism (Chen R. et al., 2022). Engineering cofactors in organelle compartmentalization benefits the biosynthesis of terpenoids that require cofactors (Cataldo et al., 2020; Zhang et al., 2022). A large number of terpene synthases like P450s and key limiting enzymes such as *HMG1* are NADH-dependent and FADH-dependent enzymes. For instance, CrtI which converts phytoene to lycopene is also a FAD-dependent enzyme (Shen et al., 2016). FAD (H2)-dependent catalytic reactions in the cytosol may be relatively inefficient due to the lower FAD(H) concentration (Pallotta et al., 1998), as the FAD (H2) concentration in mitochondria is 20 times higher than in the cytosol of *S. cerevisiae*. Different cofactors have their unique organelle localization and construction of a cofactor biosynthesis pathway in the cytosol or targeted organelles is an alternative method for increasing terpenoid production.
